# Reasons for low uptake of a psychological intervention offered to cancer survivors with elevated depressive symptoms

**DOI:** 10.1002/pon.5029

**Published:** 2019-03-05

**Authors:** Loek J. van der Donk, K. Annika Tovote, Thera P. Links, Jan L.N. Roodenburg, Johanna C. Kluin‐Nelemans, Henriette J.G. Arts, Veronique E.M. Mul, Robert J. van Ginkel, Peter C. Baas, Christiaan Hoff, Robbert Sanderman, Joke Fleer, Maya J. Schroevers

**Affiliations:** ^1^ Department of Health Psychology University of Groningen University Medical Center Groningen Groningen the Netherlands; ^2^ Department of Endocrinology and Metabolic Diseases University of Groningen University Medical Center Groningen Groningen the Netherlands; ^3^ Department of Oral and Maxillofacial Surgery, Section of Oncology University of Groningen University Medical Center Groningen Groningen the Netherlands; ^4^ Department of Haematology, University of Groningen University Medical Center Groningen Groningen Groningen the Netherlands; ^5^ Department of Obstetrics and Gynecology, University of Groningen University Medical Center Groningen Groningen the Netherlands; ^6^ Department of Radiation Oncology, University of Groningen University Medical Center Groningen the Netherlands; ^7^ Department of Surgery, Laboratory for Translational Surgical Oncology, University of Groningen University Medical Center Groningen Groningen the Netherlands; ^8^ Department of Surgery Martini Hospital Groningen the Netherlands; ^9^ Department of Surgery Medical Center Leeuwarden Leeuwarden the Netherlands; ^10^ Department of Psychology, Health and Technology University of Twente Enschede the Netherlands

**Keywords:** cancer, cancer survivors, consecutive screening, CONSORT, depression, oncology, randomized controlled trial, recruitment, screening, screening guidelines

## Abstract

**Objective:**

In line with screening guidelines, cancer survivors were consecutively screened on depressive symptoms (as part of standard care), with those reporting elevated levels of symptoms offered psychological care as part of a trial. Because of the low uptake, no conclusions could be drawn about the interventions' efficacy. Given the trial set‐up (following screening guidelines and strict methodological quality criteria), we believe that this observational study reporting the flow of participation, reasons for and characteristics associated with nonparticipation, adds to the debate about the feasibility and efficiency of screening guidelines.

**Methods:**

Two thousand six hundred eight medium‐ to long‐term cancer survivors were consecutively screened on depressive symptoms using the Patient Health Questionnaire‐9 (PHQ‐9). Those with moderate depressive symptoms (PHQ‐9 ≥ 10) were contacted and informed about the trial. Patient flow and reasons for nonparticipation were carefully monitored.

**Results:**

One thousand thirty seven survivors (74.3%) returned the questionnaire, with 147 (7.6%) reporting moderate depressive symptoms. Of this group, 49 survivors (33.3%) were ineligible, including 26 survivors (17.7%) already receiving treatment and another 44 survivors (30.0%) reporting no need for treatment. Only 25 survivors (1.0%) participated in the trial.

**Conclusion:**

Of the approached survivors for screening, only 1% was eligible and interested in receiving psychological care as part of our trial. Four reasons for nonparticipation were: nonresponse to screening, low levels of depressive symptoms, no need, or already receiving care. Our findings question whether to spend the limited resources in psycho‐oncological care on following screening guidelines and the efficiency of using consecutive screening for trial recruitment in cancer survivors.

## 1 INTRODUCTION

Depressive symptoms are common in cancer patients, not only shortly after diagnosis or during active treatment but also in cancer survivors.^1^, ^2^ As effective psychological interventions exist to treat these symptoms,^3^, ^4^, ^5^, ^6^ clinical guidelines currently recommend to routinely screen cancer patients on distress throughout the illness and treatment trajectory in order to detect distress and refer patients accordingly to additional care.^7^, ^8^ These recommendations still hold, even though so far no well‐conducted randomized control trials (RCTs) have demonstrated that mental health outcomes improve via these screening programs.^9^

Evidence for the efficacy on interventions has mostly been confirmed in patients in the short‐term phase and women with breast cancer, whereas less evidence is available for the efficacy of these interventions among cancer survivors.^3^, ^4^, ^5^, ^6^, ^10^, ^11^, ^12^ Therefore, the Dutch Cancer Foundation released a call in 2013 for more evidence regarding the efficacy of psychological interventions among (nonbreast) cancer survivors. Following strict high‐quality standards,^13^ including consecutively screening on depressive symptoms, we set up a multicenter RCT examining the efficacy of cognitive behavioral therapy (CBT) and mindfulness‐based cognitive therapy (MBCT) for treating depressive symptoms in cancer survivors. Because of the low trial participation, no conclusion could be drawn about the efficacy of the interventions. As a means to reflect on reasons why an RCT following high‐quality methodological standards failed to work in clinical practice, this observational study examined the reasons for nonparticipation in the RCT and the demographic and medical characteristics of depressed survivors that did (not) participate. Cancer survivors in our trial were consecutively screened on depressive symptoms as a part of standard care, as recommended by the current clinical screening guidelines^7^, ^8^ and regarded as a quality standard in setting up an RCT.^14^, ^15^ Yet, the screening procedure was not efficient (ie, resulting in low uptake). Findings of our study may therefore add to the debate regarding the feasibility and efficiency of current screening guidelines for identifying patients in need for care. Our aim is twofold^1^: to inform clinical practice about cancer survivors' levels of depressive symptoms and care needs and the use of consecutive screening^2^; to inform researchers in setting up future psychological RCTs in cancer survivors, to carefully reflect and make considerations regarding the use of consecutive and convenience sampling as a means for patient recruitment.

## 2 METHOD

### 2.1 Study design

This observational study used data collected as part of a multicenter RCT comparing MBCT and CBT with treatment as usual (TAU). For the current study, only the screening data was used. Data were collected from February 2015 until May 2017.

### 2.2 Participants

Eligibility criteria for being approached for screening were: a cancer diagnosis (except breast cancer), age between 18 to 75 years at the time of diagnosis, currently no active cancer, and completion of curative treatment 1 to 5 years ago. For trial participation, an additional eligibility criterion was the report of moderate levels of depressive symptoms (PHQ‐9 ≥ 10). Exclusion criteria for trial participation were: not being able to read and write Dutch, having psychiatric comorbidity, receiving psychological treatment for depressive symptoms (currently or less than 2 months ago) and an instable antidepressant regimen (ie, starting/changing less than 2 months ago).

### 2.3 Screening procedure

Individuals were routinely screened for depressive symptoms at departments radiotherapy, surgery, oral and maxillofacial surgery, gynecology, hematology, endocrinology, medical oncology, and colorectal surgery. Individuals received a letter from their department inviting them to complete a mood questionnaire (PHQ‐9) on paper or online and in case this score was elevated, they would be contacted. Individuals reporting elevated depressive symptoms (PHQ‐9 ≥ 10) received feedback about their elevated levels and were informed that they would receive a telephone call to discuss the depressive symptoms and a possible need for psychological support. These telephonic interviews were executed by graduate clinical psychologists or research/student assistants who had received special training, in which they made a clinical assessment of the psychological problems. Subsequently, persons were selected on eligibility (using a standardized interview to check for exclusion criteria), interest in psychological support and willingness to participate. If this was the case, they received written information about the trial, a questionnaire, an informed consent form, and a prepaid return envelope. They were asked to return a completed informed consent and questionnaire within 2 weeks. Individuals expressing interest in psychological support but who were ineligible or unwilling to participate were given advice to discuss their care needs with their medical specialist or general practitioner.

### 2.4 Variables

For screening on depressive symptoms, the Patient Health Questionnaire‐9 (PHQ‐9) was used,^16^ which is a self‐report screening tool based on the nine depression criteria according to the *Diagnostic and Statistical Manual of Mental Disorders*. Each item can be scored from 0 (*not at all*) to 3 (*nearly every day*), resulting in total scores ranging from 0 to 27, with higher scores indicating more depressive symptoms.

### 2.5 Statistical analyses

SPSS 25.0 was used for executing statistical analyses. Demographic (ie, age and gender) and cancer‐related characteristics (ie, years since diagnosis, years since treatment, cancer type, treatment type and recurrence) were calculated. Chi‐square tests and *t*‐tests compared groups (ie, respondents versus nonrespondents; depressed versus not depressed; in trial versus not in trial) on demographic and cancer‐related variables.

## 3 RESULTS

**Figure 1:**
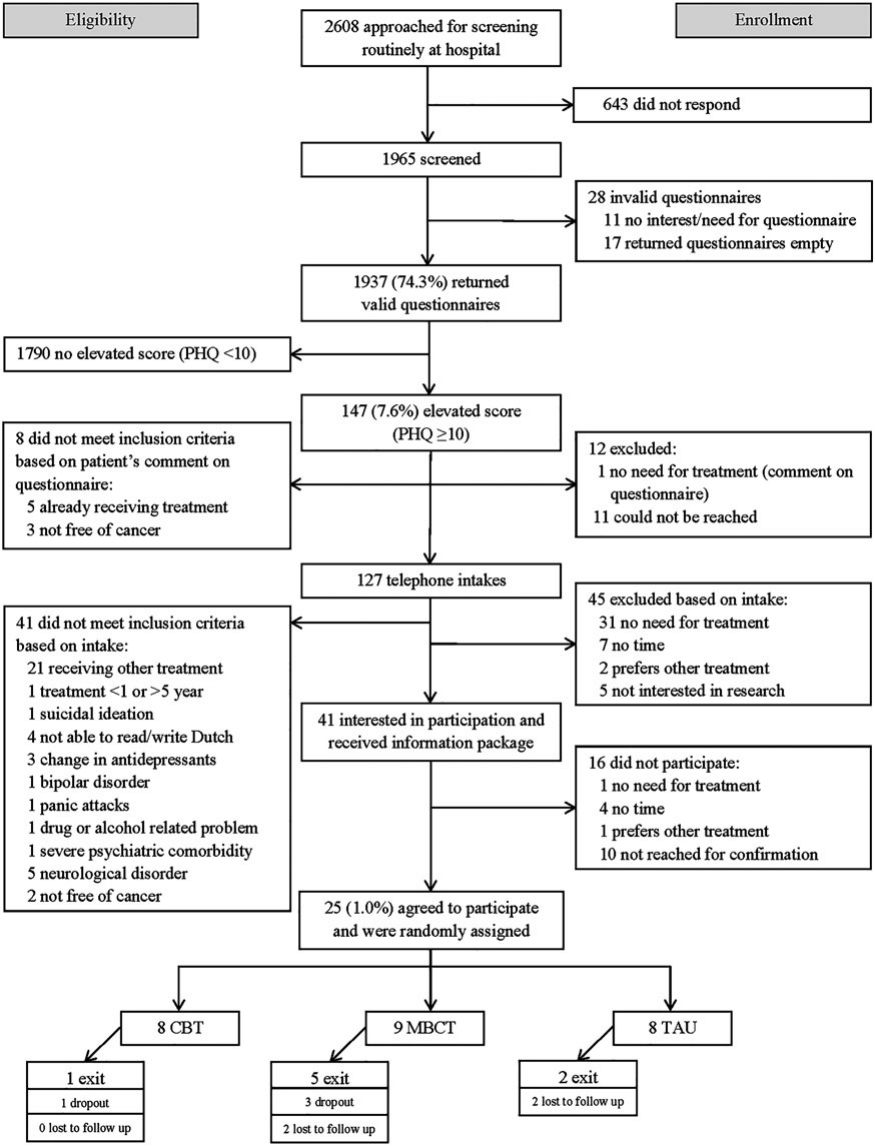
Flowchart of participant recruitment and flow through the study. PHQ, patient health questionnaire; CBT, cognitive behavioral therapy; MBCT, mindfulness‐based cognitive therapy; TAU, treatment as usual

Initially 2608 cancer survivors were invited to complete a screening questionnaire (Figure 1). In total 25 individuals agreed to participate in the RCT, which was 1.0% of the approached individuals.

Of the 2608 cancer survivors approached for routine screening, 1937 returned a valid questionnaire. Table 1 describes the demographic and cancer‐related characteristics of the 1937 cancer survivors. Mean age was 63 years with 61% being male. Average time since diagnosis and time since treatment were both 3 years. Most common cancer type was gastro‐intestinal cancer and only receiving surgery was the most common treatment. In total, 166 individuals (8.6%) reported a cancer recurrence.

**Table 1:** Demographic and medical characteristics of 1937 cancer survivors

	Cancer Survivors		
Demographic variables			
N	1937		
Age (M, SD)	63.34	10.33	
Gender, male (N, %)	1188	61.3	
Cancer‐related variables			
Years since diagnosis (M, SD)	3.40	1.31	
Years since diagnosis (N, %)			
≤ 2 y	591	30.5	
> 2 y	1346	69.5	
Years since end treatment (M, SD)	3.07	1.24	
Years since end treatment (N, %)			
1 y	218	11.3	
2 y	523	27.0	
3 y	468	24.2	
4 y	397	20.5	
5 y	330	17.0	
Cancer type (N, %)			
Lung	70	3.6	
Skin	35	1.8	
Head and neck	235	12.1	
Endocrine	59	3.0	
Gastro‐intestinal	789	40.7	
Urological	358	18.5	
Gynecological	179	9.2	
Bone & soft tissue	43	2.2	
Hematological	160	8.3	
Other/primary unknown	9	0.5	
Received treatment (N, %)			
Surgery	429	22.2	
Surgery + RT	372	19.2	
Surgery + chemotherapy	180	9.3	
Surgery + RT + chemotherapy	211	10.9	
RT	385	19.9	
RT + chemotherapy	196	10.1	
RT + hormone therapy	90	4.6	
Chemotherapy	21	1.1	
Other	52	2.7	
Recurrence (N, %)			
No	1771	91.4	
Yes	166	8.6	

Abbreviation: RT, radiation therapy. Numbers may slightly differ because of missing variables.

Those 1937 persons who returned the questionnaire were compared with those who did not return it. Compared with those who did not return the questionnaire, cancer survivors returning the questionnaire were significantly older (63.3 years ±10.3 versus 59.4 years ±13.0), more often male (61% versus 53%) and had more often a cancer recurrence (8.6% versus 4.8%). No significant differences were found in years since diagnosis or years since treatment. Concerning cancer site, highest response rates were found among survivors with bone and soft tissue (91.5%) and survivors with urological cancer (88.4%) with lowest response rates among lung cancer survivors (65.4%). A full overview regarding response rates and elevated depressive symptoms (PHQ‐9 ≥ 10) according to demographic and cancer‐related characteristics can be found in the Appendix.

In total, 147 persons reported moderate levels of depressive symptoms (PHQ ≥ 10) and these persons were compared with those 1790 persons not depressed. Those depressed were significantly younger (63.7 ± 10.1 versus 59.3 ± 11.9) compared with those not depressed. No significant differences between those survivors with or without moderate levels of depressive symptoms were found for gender, year since diagnosis, year since treatment, and cancer recurrence. Highest levels of depressive symptoms were found among lung cancer survivors (17.1%) and lowest levels of depressive symptoms among gastrointestinal cancer survivors (3.9%).

Table 2 describes a comparison between 122 individuals with elevated levels of depressive symptoms not included in the trial versus 25 individuals with elevated levels of depressive symptoms who participated in the trial. No significant differences were found between these groups on age, gender, depressive symptoms, time since diagnosis, time since treatment, or cancer recurrence.

**Table 2:** Characteristics of individuals participating in the RCT compared with those with elevated depressive symptoms that did not participate in the trial

	Depressed, Not In Trial	Depressed, In Trial	Total	*P* value
N (%)	122 (83.0%)	25 (17.0%)	147 (100%)	
Age (M, SD)	59.93 ± 11.86	56.16 ± 11.71	59.29 ± 11.88	0.149
Gender (% male)	54.90	56.00	55.10	0.921
Depressive symptoms (M, SD)	14.42 ± 3.83	13.92 ± 3.67	14.33 ± 3.80	0.552
Time since diagnosis	3.38 ± 1.22	3.75 ± 1.25	3.45 ± 1.23	0.179
Time since treatment	3.03 ± 1.16	3.09 ± 1.07	3.04 ± 1.14	0.829
Recurrence (% yes)	10.70	20.00	12.20	0.194

### 3.1 Reasons for nonparticipation

Four major reasons for nonparticipation were identified. The first reason was not responding to the screening questionnaire, with 671 persons (25.7% of 2608 cancer survivors) not returning a valid questionnaire. Secondly, low rates of depressive symptoms were observed, with only 147 persons (ie, 7.6% of those completing screening) scoring moderate levels of depressive symptoms. A third reason for nonparticipation involved low care needs, with 44 depressed persons (29.9% of 147) reporting no need or time for psychological care. A final reason for not being able to participate was already receiving treatment, reported by 26 depressed persons (17.7% of 147).

## 4 DISCUSSION

As part of an RCT, we screened a large group of cancer survivors on depressive symptoms, with those reporting moderate or higher levels of depressive symptoms being contacted to discuss their need for care, and inform them about the possibility to receive psychological care, as part of an intervention study. We encountered a very low participation rate. The current paper examined the reasons for not participating, as we believe this will provide more insight into the feasibility of routinely screening for depressive symptoms in cancer survivors as well as of the use of consecutive screening for recruiting cancer survivors for a psychological RCT. Of the 2608 survivors approached, only 7.6% reported moderate levels of depressive symptoms, and of those, almost 50% reported no psychological care needs or already received treatment.

A key finding is that most cancer survivors reported no or only mild levels of depressive symptoms (taking into account that we excluded survivors of breast cancer who are known to be a group at risk for depressive symptoms^17^, ^18^). Another main finding is that many survivors reporting elevated depressive symptoms were not interested in receiving psychological care. Our findings question whether consecutive screening on depressive symptoms as part of standard clinical practice (as recommended by clinical guidelines as well as research recommendations for recruiting trial participants)^7^, ^8^, ^14^ is feasible among cancer survivors and an efficient way to detect those with a need for care and referral. Four major reasons for nonparticipation were identified^1^: one in four cancer survivors did not return the screening questionnaire,^2^ rates of depressive symptoms were lower than expected according to literature,^3^ one in three depressed cancer survivors did not wish to receive psychological care, and^4^ a group of depressed cancer survivors already found psychological help themselves.

One in four cancer survivors could not be screened on depressive symptoms, a response rate of 75% that can be considered high when using a survey^19^ and which is also somewhat higher than response rates in other screening studies (varying from 63% to 68%) among cancer patients using surveys.^20^, ^21^, ^22^, ^23^ Research has shown that patients not responding to a screening questionnaire are also more likely to not show up for medical check‐ups, suggesting that these patients may in general be difficult to reach.^24^ An explanation for the nonresponse to screening may be the information given in the accompanied letter, using words like “depressive symptoms” and informing patients that they would be contacted in case an elevated score was reported (See Appendix).

The screening identified only a small group of cancer survivors (7.6%) reporting moderate levels of depressive symptoms. This suggests that most cancer survivors are able to adapt and do not experience depressive symptoms in the years following curative treatment. When comparing rates of depressive symptoms in cancer patients, heterogeneity in prevalence rates can be observed, related, among others, to differences in cancer type, time since diagnosis, and the specific screening instrument.^1^ Regarding cancer type, two reviews concluded that women with breast cancer are at risk for depressive symptoms,^17^, ^18^ which could explain why rates in our study were lower than expected, as women with breast cancer were not approached. In fact, most cancer survivors in our study were diagnosed with gastro‐intestinal or urological cancer, which have been associated with lower levels of depressive symptoms.^17^, ^20^ Related to this, in contrast to most previous research focusing on female survivors,^1^, ^17^, ^18^, ^25^ more than half (61%) of our sample were men that received only surgery. It has been shown that male cancer survivors have lower levels of depressive symptoms compared with women,^21^ and it can be argued that because of a good prognosis and advances in targeted cancer treatment, the impact of cancer treatment may have been reduced throughout the years, which could also have resulted in relatively low levels of depressive symptoms.^26^ Additionally, psychosocial support throughout the cancer trajectory has improved and cancer survivors in our study have possibly received intensive psychosocial support during cancer diagnosis and active treatment.

Concerning time since diagnosis, two meta‐analyses among cancer patients found depressive symptoms to decrease over time, varying from 27% (in the acute phase) to 21% (within the first year post‐treatment), to 15% (at least 1 y post‐treatment), with similar levels as healthy controls after 2 years following diagnosis.^1^, ^27^ This could also explain lower rates of depressive symptoms in our study, as cancer survivors were diagnosed and completed medical treatment on average more than 3 years ago. When interpreting the above‐mentioned findings, it should be taken into account that both meta‐analyses (like meta‐analyses in general) have included a variety of screening instruments, which hampers drawing firm conclusions regarding rates of depressive symptoms. Generally, the efficacy of screening greatly depends on the timing of the screening (ie, phase of the cancer trajectory). In our study, we targeted medium‐ to long‐term cancer survivors for screening, but if recently diagnosed cancer patients or those in active treatment would have been approached, efficacy of screening may have been higher (because of higher rates of depressive symptoms and greater uptake).

Another factor that may explain variation in rates of depressive symptoms is the measurement of symptoms, which includes the use of a clinical diagnostic interview to classify major depressive disorder versus self‐report screening questionnaires.^1^, ^28^ Although screening questionnaires are often used because of their convenience (ie, inexpensive and quick to administer to large groups), it should be noted that screening questionnaires overestimate the prevalence of depression.^28^ In addition, variation in rates of depressive symptoms may not only be explained by using different screening instruments but also by using different cutoff thresholds within a distinct instrument for determining elevated depressive symptoms.^1^ Our study used the PHQ‐9, which is commonly used in oncology for screening on depressive symptoms,^16^, ^20^, ^21^ and using a cut‐off of greater than or equal to 10, we found moderate levels of depressive symptoms rates of 7.6%. Other studies using the same criteria found similar, slightly higher percentages (9.3%‐11.3%) for a mixed group of survivors.^20^, ^25^ On the other hand, some studies label mild depressive symptoms as being depressed. Therefore, caution is warranted when comparing different rates across studies and we recommend that future research includes a precise description of what their rates of depressive symptoms refer to.

A third reason was low need for professional psychological care among depressed cancer survivors. It is worth mentioning that in our trial, care need was distinguished from willingness to participate in the trial by asking this in separate questions. In our study, almost one in three depressed persons reported no need or time for treatment. Several studies have drawn similar conclusions that cancer patients with elevated symptoms did not want a referral.^29^, ^30^, ^31^, ^32^ Possible reasons that have been identified include patients' desire to manage problems on their own^31^, ^33^, ^34^ or by means of informal social support,^30^, ^31^, ^33^ which may be related to fear of stigmatization for visiting a psychologist.^30^, ^33^ Other reasons include that depressive symptoms are not perceived as a severe burden for which professional help is warranted^34^ or the preference for receiving medication (eg, antidepressants). Although there is evidence suggesting that patients with a medical diagnosis prefer psychological treatment to antidepressant medication,^35^ more research is needed to examine cancer survivors' perceptions of and coping with depressive symptoms, their care needs, and barriers to seek care in order to identify ways to improve psycho‐oncological care.

A fourth reason was that cancer survivors already found professional psychological help themselves. In our study, this was 17.7%, and similar percentages were reported by another Dutch trial among cancer survivors 1 year after treatment^29^ and somewhat higher numbers (24%) by an Australian study on care needs in distressed cancer patients.^34^ On the other hand, three Scottish high‐quality RCTs found few depressed cancer patients to be already in treatment, varying from 0.8% to 7.0%.^36^, ^37^, ^38^ A possible explanation for the relatively high percentage of individuals already receiving treatment, as well as the low care needs in our study, can be differences in healthcare policies between countries in terms of insurance and coverage of psychosocial aftercare for cancer survivors. For instance, in the Netherlands, this is mostly covered by the insurance, making psychological care accessible for anyone irrespective of trial participation. This could explain why individuals in our trial reported low care needs and why the percentage of individuals already receiving treatment was substantial.

Currently, screening is recommended in clinical practice^7^, ^8^ as well as for trial recruitment,^15^ but in our trial screening (which was part of standard care) proved little effective in terms of detecting individuals with care needs. Only 1.0% of the approached individuals participated in the RCT. Several other trials on psychological outcomes in oncology also found low inclusion rates between 2.5% and 3.5%.^29^, ^36^ Above‐mentioned trials and our trial used consecutive sampling for patient recruitment, which encompasses systematically screening every individual who meets the selection criteria.^14^ Another frequently used sampling method involves convenience sampling in which individuals are recruited by means of (self)referral, which has advantages in terms of cost, time, and logistics, but may produce an unrepresentative sample.^14^ For this reason, consecutive sampling is generally seen as the golden standard and is favorable to convenience sampling, because the latter is more prone to selection bias.^14^ However, in practice, this may not completely be the case, because a recent trial found that consecutive sampling still resulted in considerable selection bias in terms of enrolling predominantly young and highly educated patients.^29^ Moreover, consecutive sampling is not mandated in the CONSORT guidelines (recommendations for high‐quality reporting of RCTs in order to maintain high internal validity^39^) implying that consecutive sampling is not a preferred method to convenience sampling for trial recruitment. Furthermore, convenience sampling may result in general in higher motivation among participants because of the self‐referral method.^40^ Given these considerations and our finding that most cancer survivors were not depressed and those that were did not want or already found help, it can be debated whether the methodological advantages of consecutive sampling outweigh its time and resource‐consuming procedures.^40^ We do not presume either consecutive or convenience sampling to be a superior method, but instead recommend that in the future the trial's aims and objectives should be decisive for choosing the appropriate sampling method.

### 4.1 Study limitations

Findings of our study need to be set in the context of several limitations. The first is that no information is available for nonresponders regarding depression, so our findings can only be generalized to those returning the questionnaire. Possibly among nonresponders, there were depressed individuals that would have influenced rates of depressive symptoms. Another limitation was the self‐report measure of depressive symptoms, which may have resulted in not depressed individuals (ie, false‐positives) being contacted or that false‐negatives were not approached for help.

### 4.2 Clinical implications

Our findings suggest that screening cancer survivors consecutively on depressive symptoms as part of standard care was not effective for recruitment in a psychological trial. Of the initially approached cancer survivors, 99% was ineligible, unwilling to participate, or could not be reached. Major reasons for nonparticipation included nonresponse to screening, low rates of depressive symptoms, low care needs, or already receiving psychological treatment. Overall, given the minimal gain from routine screening as suggested by our findings as well as previous research,^9^ it can be questioned whether the required resources would seem better spent on providing inexpensive or free resources to those who need them or on providing psychological education to patients. These findings should be considered when designing future psychological trials in cancer survivors or when screening (for patient recruitment) is considered.

## CONFLICT OF INTEREST

The authors have no potential conflicts of interest to report.

## ETHICS STATEMENT

The study was approved by the Medical Ethical Committee of the University Medical Center Groningen (METc 2014/214).

## APPENDIX 1

### A.1. Response rates and elevated depressive symptoms (PHQ‐9 ≥ 10) according to demographic and cancer‐related characteristics

**Table:**

Characteristic	Response Rate, %	Elevated Score^c^, %
Age^a^		
<57	62.6	12.2
57‐66	75.8	9.4
66‐70	80.4	4.6
>70	78.0	5.1
Gender		
Male	76.8	6.8
Female	70.5	8.8
Cancer type		
Lung	65.4	17.1
Skin	77.8	8.6
Head and neck	78.6	12.3
Endocrine	73.8	11.9
Gastro‐intestinal	76.2	3.9
Urological	88.4	7.8
Gynecological	68.6	8.9
Bone & soft tissue	91.5	7.0
Hematological	75.8	10.0
Other/primary unknown	81.8	22.2
Treatment type		
Surgery	97.9	4.9
Surgery + RT^b^	81.6	7.0
Surgery + chemotherapy	98.4	3.9
Surgery + RT^b^ + chemotherapy	81.5	4.7
RT^b^	79.1	12.2
RT^b^ + chemotherapy	74.0	11.2
RT^b^ + hormone therapy	85.7	7.8
Chemotherapy	100.0	19.0
Other	98.1	5.8
Time since diagnosis		
Less than 2.5 y	88.5	6.4
More than 2.5 y	84.1	8.1
Time since treatment		
1 y	92.0	6.4
2 y	86.7	7.5
3 y	83.6	9.4
4 y	81.7	7.6
5 y	86.6	6.1
Cancer recurrence		
No	84.9	7.3
Yes	91.2	10.8

a Categories were based on quartiles.

b RT = Radiotherapy Treatment.

c Determined by PHQ‐9

### A.2. Screening letter for patients that was attached to the screening questionnaire

Dear [MISS/SIR],

You are in follow‐up at our department because you have had cancer in the past. Whenever you visit our hospital for a medical check‐up, our main aim is to find out how you are doing in terms of medical health. Research, however, has shown that a diagnosis of cancer and treatment can cause feelings of tension, sadness and insecurity and that these emotional complaints can persist for a long while after cancer treatment has finished.

**Questionnaire**

Our department considers it important to also give attention to the emotional consequences of having had cancer. For this reason, a short questionnaire has been developed with questions regarding your current mood. You can fill in this questionnaire within five minutes at home via the internet. If you do not have internet access or if you encounter other problems when filling in the questionnaire, you can also make use of the attached paper questionnaire and send this back using the prepaid return envelope (a stamp is not required).

To fill in the online questionnaire at home, you can visit:

[WEBSITE]

In the questionnaire, you will be asked about your security code. Your personal security code is:

[SECURITY CODE]

**Results**

If the results from the questionnaire indicate that you have, for instance, depressed or tensed feelings, you will be contacted. The result of the questionnaire will also be in your medical records, making the information also accessible for your medical practitioner. Therefore, you can, if you want to, discuss the results of the questionnaire with your medical practitioner. You can call us as well if you have any questions. [PHONE NUMBER]

We would like to thank you in advance for your cooperation.

Kind regards,

[NAME]

## References

[1] Krebber AMH , Buffart LM , Kleijn G , et al. Prevalence of depression in cancer patients: a meta‐analysis of diagnostic interviews and self‐report instruments. Psychooncology. 2014;23(2):121‐130. 24105788 10.1002/pon.3409 PMC4282549

[2] Bower JE . Behavioral symptoms in patients with breast cancer and survivors. J Clin Oncol [Internet. 2008;26(5):768‐777. Available from: 10.1200/JCO.2007.14.3248 18258985 PMC3057774

[3] Galway K , Black A , Cantwell M , Cr C , Mills M , Donnelly M . Psychosocial interventions to improve quality of life and emotional wellbeing for recently diagnosed cancer patients. Cochrane Database of Syst Rev. 2012;(11):CD007064. 10.1002/14651858.CD007064.pub2 PMC6457819 23152241

[4] Faller H , Schuler M , Richard M , Heckl U , Weis J , Ku R . Effects of psycho‐oncologic interventions on emotional distress and quality of life in adult patients with cancer: systematic review and meta‐analysis. J Clin Oncol. 2017;31(6):782‐793. 10.1200/JCO.2011.40.8922 23319686

[5] Jacobsen PB , Jim HS . Psychosocial interventions for anxiety and depression in adult cancer patients: achievements and challenges. CA ‐ A Cancer J Clin. 2008;58(4):214‐230. 10.3322/CA.2008.0003 18558664

[6] Williams S , Dale J . The effectiveness of treatment for depression/depressive symptoms in adults with cancer: a systematic review clinical studies 2006;372–390. 10.1038/sj.bjc.6602949 PMC2361139 16465173

[7] Carlson LE , Waller A , Mitchell AJ . Screening for distress and unmet needs in patients with cancer: review and recommendations. J Clin Oncol. 2012;30(11):1160‐1177. 22412146 10.1200/JCO.2011.39.5509

[8] National Institutes Panel HS . National Institutes of Health state‐of‐the‐science conference statement: symptom management in cancer: pain, depression, and fatigue, July 15‐17, 2002. J Natl Cancer Inst Monogr [Internet]. 2004;2004(32):9‐16. Available from: https://academic.oup.com/jncimono/article‐lookup/doi/10.1093/jncimonographs/djg014 10.1093/jncimonographs/djg014 15263035

[9] Meijer A , Roseman M , Milette K , et al. Depression screening and patient outcomes in cancer: a systematic review. PLoS ONE. 2011;6(11):e27181. 22110613 10.1371/journal.pone.0027181 PMC3215716

[10] Li M , Fitzgerald P , Rodin G . Evidence‐based treatment of depression in patients with cancer. 2017;30(11):1187‐1196. 10.1200/JCO.2011.39.7372 22412144

[11] Luckett T , Britton B , Clover K , Rankin NM . Evidence for interventions to improve psychological outcomes in people with head and neck cancer: a systematic review of the literature. Support Care Cancer. 2011;19(7):871‐881. 21369722 10.1007/s00520-011-1119-7

[12] Walker J , Sawhney A , Hansen CH , et al. Treatment of depression in adults with cancer: a systematic review of randomized controlled trials. Psychol Med. 2014;2017:897‐907. 10.1017/S0033291713001372 23778105

[13] Higgins JPT , Altman DG , Sterne JAC . Chapter 8: Assessing risk of bias in included studies. In: Higgins JPT, Green S (eds). Cochrane Handbook for Systematic Reviews of Interventions. Version 5.1.0 (updated March 2011). The Cochrane Collaboration, 2011 Available from www.handbook.cochrane.org.

[14] Kendall J . Designing a research project: randomised controlled trials and their principles. Emerg Med J. 2003;2003:164‐169. 10.1136/emj.20.2.164 PMC1726034 12642531

[15] Sanjida S , Mcphail SM , Shaw J , et al. Are psychological interventions effective on anxiety in cancer patients? A systematic review and meta‐analyses. Psychooncology. 2018;27:2063‐2076. 10.1002/pon.47942076 29885258

[16] Kroenke K , Spitzer RL , Williams JBW . The PHQ‐9: validity of a brief depression severity measure. J Gen Intern Med. 2001;16(9):606‐613. 11556941 10.1046/j.1525-1497.2001.016009606.x PMC1495268

[17] Massie MJ . Prevalence of depression in patients with cancer. J Natl Cancer Inst Monogr [Internet]. 2004;(32):57‐71. Available from: http://www.ncbi.nlm.nih.gov/entrez/query.fcgi?cmd=Retrieve&db=PubMed&dopt=Citation&list_uids=15263042 10.1093/jncimonographs/lgh014 15263042

[18] Fann JR , Thomas‐Rich AM , Katon WJ , et al. Major depression after breast cancer: a review of epidemiology and treatment. Gen Hosp Psychiatry. 2008;30(2):112‐126. 18291293 10.1016/j.genhosppsych.2007.10.008

[19] Van Horn PS , Green KE , Martinussen M . Survey response rates and survey administration in counseling and clinical psychology: a meta‐analysis. Educ Psychol Meas. 2009;69(3):389‐403.

[20] Hinz A , Mehnert A , Kocalevent R , et al. Assessment of depression severity with the PHQ‐9 in cancer patients and in the general population. BMC Psychiatry [Internet]. 2016;16(22):1‐8. Available from: 10.1186/s12888-016-0728-6 PMC4736493 26831145

[21] Hartung TJ , Brähler E , Faller H , et al. The risk of being depressed is significantly higher in cancer patients than in the general population: prevalence and severity of depressive symptoms across major cancer types. Eur J Cancer. 2017;72(2017):46‐53. 28024266 10.1016/j.ejca.2016.11.017

[22] Hartung TJ , Friedrich M , Johansen C , et al. The Hospital Anxiety and Depression Scale (HADS) and the 9‐item Patient Health Questionnaire (PHQ‐9) as screening instruments for depression in patients with cancer. Cancer [Internet. 2017; Available from: 10.1002/cncr.30846;123(21):4236‐4243. 28654189

[23] Mehnert A , Koch U . Psychological comorbidity and health‐related quality of life and its association with awareness, utilization, and need for psychosocial support in a cancer register‐based sample of long‐term breast cancer survivors. J Psychosom Res. 2008;64(4):383‐391. 18374737 10.1016/j.jpsychores.2007.12.005

[24] Fleer J , Tovote KA , Keers JC , et al. Screening for depression and diabetes‐related distress in a diabetes outpatient clinic. Diabet Med. 2013;30(1):88‐94. 22924587 10.1111/dme.12001

[25] Bevilacqua LA , Dulak D , Schofield E , et al. Prevalence and predictors of depression, pain, and fatigue in older‐ versus younger‐adult cancer survivors. Psychooncology. 2018;27:900‐907. 10.1002/pon.4605 29239060 PMC7343079

[26] DeSantis CE , Lin CC , Mariotto AB , et al. Cancer treatment and survivorship statistics, 2014. CA Cancer J Clin. 2014;64(4):252‐271. 24890451 10.3322/caac.21235

[27] Mitchell AJ , Ferguson DW , Gill J , Paul J , Symonds P . Depression and anxiety in long‐term cancer survivors compared with spouses and healthy controls: a systematic review and meta‐analysis. Lancet Oncol [Internet]. 2013;14(8):721‐732. Available from: 10.1016/S1470-2045(13)70244-4 23759376

[28] Thombs BD , Kwakkenbos L , Levis AW , Benedetti A . Addressing overestimation of the prevalence of depression based on self‐report screening questionnaires. Cmaj [Internet. 2018;190(2):E44‐E49. Available from: 10.1503/cmaj.170691 29335262 PMC5770251

[29] Van Scheppingen C , Schroevers MJ , Pool G , et al. Is implementing screening for distress an efficient means to recruit patients to a psychological intervention trial? Psychooncology. 2014;23(5):516‐523. 24829951 10.1002/pon.3447

[30] Admiraal JM , van Nuenen FM , Burgerhof JGM , Reyners AKL , Hoekstra‐Weebers JEHM . Cancer patients' referral wish: effects of distress, problems, socio‐demographic and illness‐related variables and social support sufficiency. Psychooncology. 2016;25(11):1363‐1370. Available from: 10.1002/pon.4067 26804486

[31] Van Scheppingen C , Schroevers MJ , Smink A , et al. does screening for distress efficiently uncover meetable unmet needs in cancer patients. Psychooncology [Internet. 2011;20(6):655‐663. Available from: http://www.ncbi.nlm.nih.gov/pubmed/22412146 21381148 10.1002/pon.1939

[32] Tuinman MA , Gazendam‐Donofrio SM , Hoekstra‐Weebers JE . Screening and referral for psychosocial distress in oncologic practice: use of the distress thermometer. Cancer. 2008;113(4):870‐878. 18618581 10.1002/cncr.23622

[33] Baker‐Glenn EA , Park B , Granger L , Symonds P , Mitchell AJ . Desire for psychological support in cancer patients with depression or distress: validation of a simple help question. Psychooncology. 2011;20(5):525‐531. 20878852 10.1002/pon.1759

[34] Clover KA , Mitchell AJ , Britton B , Carter G . Why do oncology outpatients who report emotional distress decline help? Psychooncology. 2014;818(December 2014:812‐818. 10.1002/pon.3729 25504987

[35] Dwight‐Johnson M , Sherbourne CD , Liao D , Wells KB . Treatment preferences among depressed primary care patients. J Gen Intern Med. 2000;1:527‐534. 10.1046/j.1525-1497.2000.08035.x PMC1495573 10940143

[36] Strong V , Waters R , Hibberd C , et al. Management of depression for people with cancer (SMaRT oncology 1): a randomised trial. Lancet. 2008;372(9632):40‐48. 18603157 10.1016/S0140-6736(08)60991-5

[37] Sharpe M , Walker J , Hansen CH , et al. Integrated collaborative care for comorbid major depression in patients with cancer (SMaRT Oncology‐2): a multicentre randomised controlled eff ectiveness trial. Lancet [Internet]. 2014;384(9948):1099‐1108. Available from: 10.1016/S0140-6736(14)61231-9 25175478

[38] Walker J , Hansen CH , Martin P , et al. Integrated collaborative care for major depression comorbid with a poor prognosis cancer (SMaRT Oncology‐3): a multicentre randomised controlled trial in patients with lung cancer. Lancet Oncol [Internet]. 2014;15(10):1168‐1176. Available from: 10.1016/S1470-2045(14)70343-2 25175097

[39] Altman DG , Schulz KF , Moher D , et al. Academia and clinic the revised CONSORT statement for reporting randomized trials. Ann Intern Med [Internet. 2001;134(8):663‐694. Available from: http://www.ncbi.nlm.nih.gov/pubmed/11304107 11304107 10.7326/0003-4819-134-8-200104170-00012

[40] Thewes B , Rietjens JAC , van den Berg SW , et al. One way or another: the opportunities and pitfalls of self‐referral and consecutive sampling as recruitment strategies for psycho‐oncology intervention trials. Psychooncology. 2018;27(8):2056‐2059. 29808508 10.1002/pon.4780 PMC6100450

